# Clinical efficacy of recombinant human soluble thrombomodulin in patients with septic disseminated intravascular coagulation

**DOI:** 10.1186/cc11997

**Published:** 2013-03-19

**Authors:** Y Suzuki, R Sato, M Sato, S Endo

**Affiliations:** 1 Iwate Medical University, Morioka, Japan

## Introduction

Thrombomodulin is an endothelial cell cofactor and glycoprotein for thrombin-catalyzed activation of protein C. A recombinant human soluble thrombomodulin (rhsTM) has been recently developed, and this new agent has a unique amino-terminal structure exhibiting anti-inflammatory activity including sequestraction and cleavage of high-mobility group box 1(HMGB-1).

## Methods

In this study, 13 patients with septic disseminated intravascular coagulation (DIC) were treated with rhsTM, which is Recomodulin^® ^Inj. 12800 (Asahi Kasei Pharma Co., Tokyo, Japan). Patients with septic DIC were treated with 130 to 3803 U/kg/day.

## Results

There were significant results for improvement of APACHE II score and DIC diagnostic criteria score for critically ill patients after treatment using rhsTM (*P <*0.01). Improvement for platelet count and D-dimer level were also observed in this study (*P <*0.05). Activation of antithrombin (AT) also was significantly increased after treatment (*P <*0.05). Hospital mortality was 15.4% in this study.

## Conclusion

The rhsTM might be one of most important endogenous regulators of coagulation, acting as the major inhibitor of thrombin as well as AT III. This new agent may play an important role in treatment for septic DIC.

